# An asymmetric producer-scrounger game: body size and the social foraging behavior of coho salmon

**DOI:** 10.1007/s12080-018-0375-2

**Published:** 2018-05-01

**Authors:** Jessica A. Phillips, Stephanie J. Peacock, Andrew Bateman, Mackenzie Bartlett, Mark A. Lewis, Martin Krkošek

**Affiliations:** 1 Department of Ecology and Evolutionary Biology, University of Toronto, Toronto, Canada; 2 Department of Zoology, University of Oxford, Oxford, UK; 3 Department of Biological Sciences, University of Alberta, Edmonton, Canada; 4 Present Address: Department of Biological Sciences, University of Calgary, Calgary, Canada; 5 Salmon Coast Field Station, Simoom Sound, Echo Bay, Canada; 6 Department of Mathematical and Statistical Sciences, University of Alberta, Edmonton, Canada

**Keywords:** Kleptoparasitism, Game theory, Coho salmon, Social foraging

## Abstract

A tension between cooperation and conflict characterizes the behavioral dynamics of many social species. The foraging benefits of group living include increased efficiency and reduced need for vigilance, but social foraging can also encourage theft of captured prey from conspecifics. The payoffs of stealing prey from others (scrounging) versus capturing prey (producing) may depend not only on the frequency of each foraging strategy in the group but also on an individual’s ability to steal. By observing the foraging behavior of juvenile coho salmon (*Oncorhynchus kisutch*), we found that, within a group, relatively smaller coho acted primarily as producers and took longer to handle prey, and were therefore more likely to be targeted by scroungers than relatively larger coho. Further, our observations suggest that the frequency of scrounging may be higher when groups contained individuals of different sizes. Based on these observations, we developed a model of phenotype-limited producer-scrounger dynamics, in which rates of stealing were structured by the relative size of producers and scroungers within the foraging group. Model simulations show that when the success of stealing is positively related to body size, relatively large predators should tend to be scroungers while smaller predators should be producers. Contrary to previous models, we also found that, under certain conditions, producer and scrounger strategies could coexist for both large and small phenotypes. Large scroungers tended to receive the highest payoff, suggesting that producer-scrounger dynamics may result in an uneven distribution of benefits among group members that—under the right conditions—could entrench social positions of dominance.

**Electronic supplementary material:**

The online version of this article (10.1007/s12080-018-0375-2) contains supplementary material, which is available to authorized users.

## Introduction

Group living has many potential benefits, including reduced predation risk and increased foraging success (Evans et al. 2015), but natural selection can produce cheaters that parasitize other group members (Nowak 2006). One example of social parasitism is kleptoparasitism, where group members steal food from others rather than foraging for themselves (Brockmann and Barnard 1979). Kleptoparasitism is perhaps one of the most widespread forms of intraspecific exploitation in the animal world and is common among birds (Brockmann and Barnard 1979; Hansen 1986; Temeles 1990; Morand-Ferron et al. 2007; Kane et al. 2014), invertebrates (Whitehouse 1997; Agnarsson 2002), mammals (Gorman et al. 1998), and fish (Nilsson and Brönmark 1999; Dill and Davis 2012).

In foraging groups subject to kleptoparasitism, individuals can act as producers that search for and capture prey or as scroungers that steal prey from other group members (Barnard 1984). In some systems, individuals can search for food while simultaneously looking for scrounging opportunities and “join in” when food is found by any individual in the group. This is known as the information-sharing model of kleptoparasitism, and in this case, scrounging does not affect the overall foraging success of the group. However, if producing and scrounging are mutually exclusive strategies such that individuals can only act as a producer or a scrounger at any given moment, then producer-scrounger dynamics can be modeled as an alternative-option game (Vickery et al. 1991; Barta and Giraldeau 1998). In this case, individuals acting as scroungers cannot bring new prey into the group, and, thus, overall group foraging success invariably declines with an increase in the proportion of scroungers in the group (Giraldeau and Caraco 2000). This results in frequency-dependent success of the scrounging strategy: at low frequency, scroungers’ payoff is high, but if all individuals in a group are scroungers, then their payoff is zero.

The frequency dependence of payoffs leads to an equilibrium frequency of scroungers in a foraging group where the payoffs to producers and scroungers are equal. In terms of game theoretic dynamics, this equilibrium frequency is the Nash equilibrium at which an individual’s payoff cannot be increased by changing strategy, provided the strategies assumed by other group members remain fixed (Giraldeau and Caraco 2000; Apaloo et al. 2014). Importantly, this Nash equilibrium does not necessarily involve a mixture of producers and scroungers; depending on the parameters of the game, the Nash equilibrium may occur in a group consisting entirely of producers.

Games in which individuals in the group differ in their foraging abilities are known as phenotype-limited or asymmetric games (Giraldeau and Beauchamp 1999), and are likely common in ecological systems where heterogeneity can arise from many factors, such as age, sex, and body size. For example, Hansen (1986) found that large adult bald eagles (*Haliaeetus leucocephalus*) profited more from scrounging, while smaller and younger individuals profited more from producing. In addition, bald eagle scroungers tended to select their targets for theft based on the relative size of the producers (Hansen 1986). Similarly, house sparrows (*Passer domesticus*) with higher dominance ranks in the foraging group scrounged from others more often (Liker and Barta 2002). On the other hand, empirical evidence that inefficient foragers utilize the scrounging strategy more often can be found from foraging groups of zebra finches (*Taenopygia guttata*) (Beauchamp 2006), kelp gulls (*Larus dominicanus*) (Steele and Hockey 1995), and oystercatchers (*Haematopus ostralegus*) (Goss-Custard et al. 1998).

Few models consider heterogeneity in the social foraging behavior of individuals within a group, and those that do have focused on interspecific kleptoparasitism (e.g., Kane et al. 2014), aggression in a hawk-dove game of resource defense in group foragers (Dubois et al. 2003), or use an individual-based modeling framework (Broom and Ruxton 2003; Broom et al. 2008). However, Barta and Giraldeau (1998) developed a model in which socially dominant individuals were assumed to be more competitive, with the resulting predictions that dominant individuals are more likely to be scroungers and tend to accrue higher payoffs than less-dominant conspecifics. For animals that show aggressive kleptoparasitism, where food items are taken by force and producers are left with nothing, intraspecific heterogeneity in foraging traits may be even more important than when producers keep a “finder’s share” of food items.

We developed a game-theoretic producer-scrounger model that builds on previous models, with explicit consideration of how phenotypic differences influence the rate of successful theft from other group members. Our primary goal was to understand how these phenotypic differences shape the behavioral dynamics of predator groups in general. We consider two discrete phenotypes, but explore how producer-scrounger dynamics are affected by different group sizes and compositions, as well as how different rates of stealing and consumption between phenotypes influence the Nash equilibrium of scrounging in each phenotype and consequent payoffs.

The model we present was motivated by observations of producer-scrounger behavior in juvenile coho salmon (*Oncorhynchus kisutch*) during group foraging (see video in Online Supplement), which suggested that larger fish within a group are more likely to act as scroungers than smaller fish. Thus, our secondary goal was to use the model we developed to understand the foraging behavior of coho salmon. Unlike previous models of producer-scrounger dynamics, the results suggest that producer and scrounger strategies may co-exist for both phenotypes under certain conditions.


ESM 2(MP4 32,599 kb)


## Empirical motivation

Yearling coho are primary predators of juvenile pink and chum salmon (Parker 1968), and their predatory behavior in freshwater streams has been extensively studied (e.g., Chapman 1962; Dill et al. 1981; Dill and Fraser 1984; Nielsen 1992). However, little is known about how predatory behavior changes when coho salmon follow their prey as they migrate into the coastal marine environment, where the territories established in streams break down. In the marine environment, yearling coho salmon have been shown to selectively prey on smaller individuals of a prey population (Parker 1971; Hargreaves and LeBrasseur 1986), to prefer pink salmon over chum salmon (Hargreaves and LeBrasseur 1985; Peacock et al. 2015), and to select prey that are parasitized (Krkošek et al. 2011; Peacock et al. 2015). However, the actual behavioral dynamics of coho salmon schools in the marine environment have not been reported. Our first anecdotal observations of coho salmon predation occurred during routine monitoring of juvenile pink and chum salmon (Peacock et al. 2016), when coho predators were sometimes caught as we set our beach seine and we were able to observe their predation behavior in a semi-natural condition. During these opportunistic observations, we noted the apparent “producer-scrounger” dynamics of coho salmon and therefore set out to more formally record the behavioral dynamics of schools of coho salmon predators.

### Methods

We conducted a field-based observational study in the Broughton Archipelago, British Columbia (50° 45′ N, 126° 30′ W), from April to May 2014, to investigate the foraging behavior of groups of coho salmon smolts on juvenile pink (*Oncorhynchus gorbuscha*) and chum (*Oncorhynchus keta*) salmon prey. The first part of the study consisted of 24 1-h observation trials with groups of 10, 15, or 20 predatory coho drawn from two independent cohorts of coho salmon. Due to some limitations of this study that we discuss below, we followed up with additional observations in smaller net pens where individual coho could be more easily tracked, allowing for more accurate observations of foraging behavior and predator sizes. The results of these additional observations generally agreed with our initial study, but there were a small number of trials, and results were not statistically significant. Details of these additional observations are in the Online Supplement.

Our behavioral observations were part of another study on how parasites on prey affect predator preference (Peacock et al. 2015), and therefore, some of the prey were infested with sea louse parasites (*Lepeophtheirus salmonis*). We did not find any significant differences in the size structure of predator dynamics in trials with infested prey versus predator behavior in trials with prey that were not infested (Table S1 and Fig. S1). All procedures were approved by the University of Alberta Biosciences Animal Care and Use Committee (AUP00000556), and salmon collections were made under Fisheries and Oceans Canada scientific fishing license number XR 62 2014.

#### Salmon collection and housing

We captured two cohorts of coho predators (*n =* 134 and *n* = 111) and pink and chum prey by beach seine (dimensions 9.1 m × 1.5 m deep with 4-mm mesh). Coho salmon predators were on average 122.5 mm (95% CI 120.8, 124.1) in fork length (from the anterior tip of the snout to the fork in the tail), while pink and chum salmon prey were on average 54.1 mm (53.7, 54.5) in fork length (Fig. S2). Coho salmon have been observed to consume prey up to 50% of their body length (Hargreaves and LeBrasseur 1986), so we excluded six coho under 80 mm from trials to reduce the possibility of gape limitation affecting the coho predation. Coho predators were housed in a large net pen (6.1 m × 6.1 m × 2.8 m deep) and fed mixed-species schools of pink and chum salmon prey at a rate of approximately two prey per predator per day (see video of kleptoparasitism in coho during routine feeding; Supplementary Data). This regular feeding resulted in an initial period of intense predation activity that lasted about 20 min, after which the level of activity declined but prey were still available. Thus, it appeared that food was not limiting in the holding pen and any differences in hunger levels with size of predators were probably less than would be found in the wild. Pink and chum salmon prey were collected 24–48 h prior to trials and housed in smaller flow-through ocean enclosures (1 m × 1 m × 0.5 m deep) and fed commercial salmon feed (micro #0-1; EWOS Canada, Surrey, British Columbia) at a rate of ~ 1.5% body mass per day.

#### Observational methodology

We performed 24 1-h observation periods (trials) between April 24 and May 27, 2014. Each trial involved groups of 10 (*n* = 7 trials), 15 (*n* = 2 trials), or 20 (*n* = 15 trials) coho predators. The number of predators depended on the number of suitable juvenile pink and chum salmon prey we were able to obtain on the day of the trials (between 40 and 100 prey per trial), resulting in a ratio of prey per predator from 2.4 to 6.6, with an average ratio of 4.2 among all trials. Before trials, we haphazardly selected coho predators from the large net pen. Selected coho were food deprived for 48–60 h. To allow predators and prey to acclimatize to the study environment, 4 to 16 h before each trial, we moved selected coho predators to one half of a diagonally divided, dark-green net pen (2.3 m × 3.2 m × 4.4 m deep) and placed equal numbers of pink and chum prey in the other half of the net pen.

The trial started when the divider in the pen was removed and coho predators were allowed access to prey. We recorded five different foraging behaviors: (1) strikes where the predator rapidly lunged at prey, whether or not the strike resulted in a capture; (2) successful captures of prey; (3) attempted thefts where one predator (the scrounger) rapidly lunged at the prey in another predator’s mouth but was not successful in stealing the prey; (4) successful thefts of prey; and (5) prey escapes where captured or stolen prey escaped before being consumed. Multiple observations may have been recorded for a single predator-prey interaction if, for example, a strike was successful and resulted in a capture, or a predator successfully captured a prey but then it was stolen by a conspecific. Prey tended to cluster at the surface, allowing us to observe the foraging behavior of the coho predators. For each behavior observed, at least two and up to four observers came to a consensus on the size of the predator(s) involved as small, medium, or large, relative to the other predators in the trial. For thefts, we noted both the size of the scrounger and the size of the targeted predator. When observers were unsure or could not come to a consensus on the size, the predator size was not recorded (i.e., treated as missing data). We recognize that this size classification is relative to other visible coho at the time of the observation and prone to observation error, which is why we followed up with additional observations where coho were size-sorted, and thus, size differentiation in mixed trials was clear (see Online Supplement).

After each trial, coho predators were returned to the holding pen. Due to restrictions on the number of coho salmon we were able to obtain and the limited number of net pens we had to house them, we had to re-use coho in trials; there were a total of 400 coho sampled from the pool of 245 individuals over the course of the study. We did not measure coho in each trial, but we measured the fork length of all coho at the end of the final trials with each cohort. We also had data on the sizes of selected coho from a similar study conducted in 2013 (Peacock et al. 2015), yielding a total of 11 trials for which we had data on the size distributions of selected coho. In all of these trials, the size distributions of selected coho were not significantly different from the size distribution of those that were not selected (Table S2), and thus, we assumed that the size distribution of coho predators in all trials was the same as the size distribution of the overall cohort.

#### Data analysis

We performed all data analyses in R (R Development Core Team 2016). We compared the rates per coho per hour of the different foraging behaviors among small, medium, and large coho across all trials using generalized linear mixed-effects models (GLMMs). Not all behaviors were observed for all sizes of coho predator in each trial, leading to an overabundance of zeros in the data, so we analyzed the rates (including zeros) using a Tweedie compound Poisson linear mixed model with the package cplm (Zhang 2013). We also compared the proportion of strikes that resulted in captures and the proportion of theft attempts that resulted in successful thefts among coho sizes using binomial GLMMs. In both the Tweedie and binomial models, we included nested random effects for trial within day within coho group that accounted for the non-independence of observations within a given trial (because there could be multiple observations of the same coho), between paired trials on each day (because of potential shared variation of paired trials due to weather or time in captivity of coho), and for the use of predators from the same group of coho. To assess the significance of coho size on the different outcome variables, we compared the fit of models for the rate of each behavior with and without the fixed effect of coho size using a likelihood ratio test.

### Results

The total population of predators used in the study (*n* = 245) was composed of 16% small, 55% medium, and 29% large coho predators. Given these percentages, small coho initiated a disproportionately large number of the observed strikes, captures, losses to theft, and prey escapes, while large coho accounted for a disproportionately small number of these behaviors (Table 1). From our observations, it appeared that small coho had higher rates of prey consumption (i.e., captures + successful thefts – loss to theft – loss to escape), but the rate of escapes may be underestimated due to difficulty observing at depth, which affected small coho disproportionately (Table 1).

**Table 1:** Proportion of different foraging behaviors observed for size classes of coho

Observation	Number	Proportion for size class		
		Small (*n* = 40)	Medium (*n* = 134)	Large (*n* = 71)
Number of coho available for trials	245	0.16	0.55	0.29
Strikes	830	0.44	0.50	0.05
Captures	367	0.43	0.51	0.06
Attempted thefts	109	0.26	0.57	0.17
Successful thefts	19^a^	0.16	0.47	0.37
Targeted by scroungers	109	0.64	0.34	0.02
Loss to theft	17^a^	0.76	0.24	0.00
Escape	68	0.65	0.34	0.01

^a^In two cases, the size of the scrounger in a theft was identified but not the size of the fish that lost the prey item

**Fig. 1:**
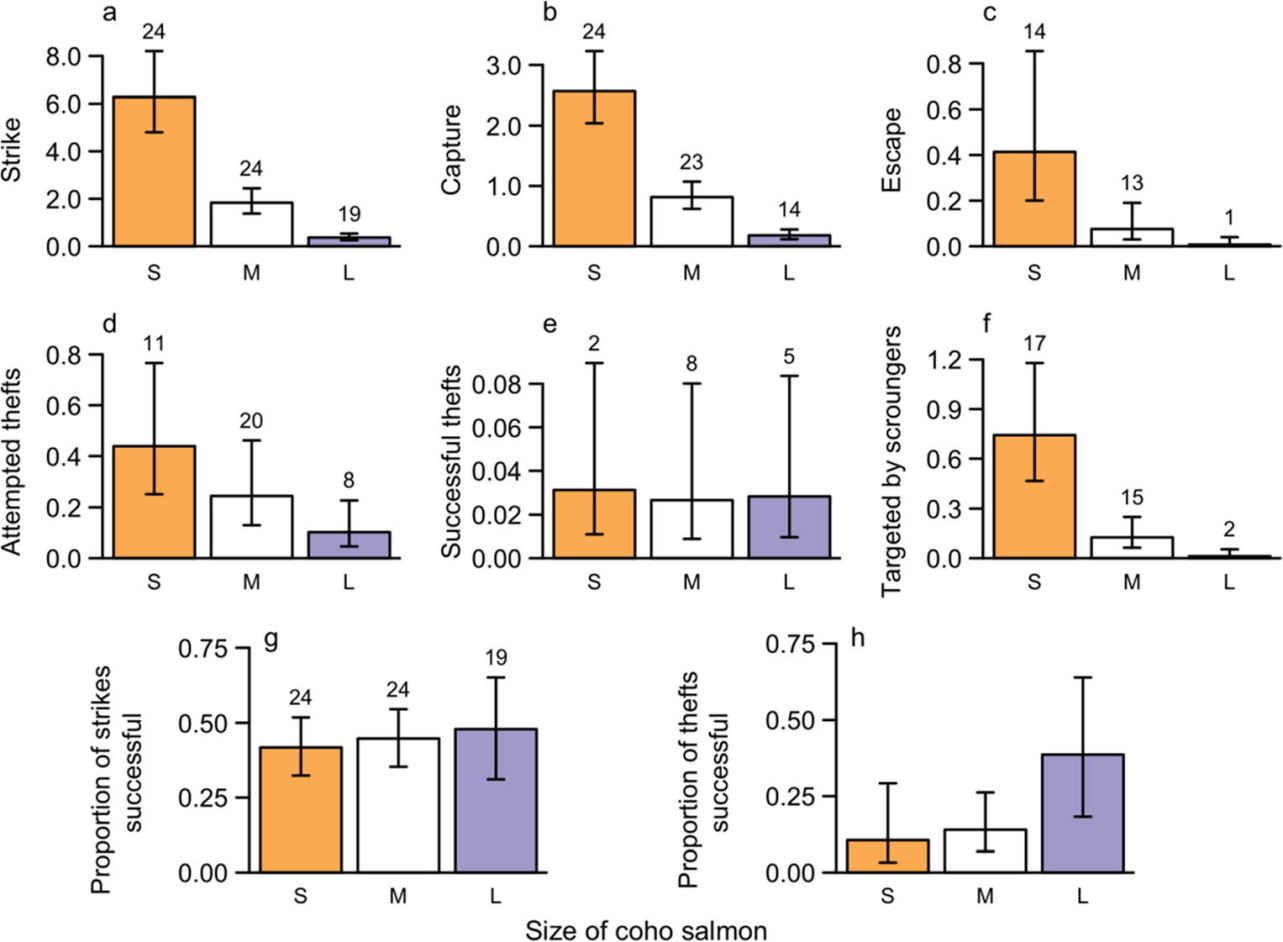
Estimated rates of coho strikes (a), captures (b), prey escapes (c), attempted thefts (d), and successful thefts (e) by coho predators and thefts on (f) coho predators in small, medium, and large size classes, and the proportion of strikes that resulted in captures (g) and proportion of attempted thefts that were successful (h) by size class. The height of the bars reflects the model estimates for the rates of each behavior per coho per hour, and the error bars show the 95% confidence intervals of the estimates. Numbers on top of bars are the number of trials (out of 24 trials) in which the given behavior was observed at least once

With the exception of the rate of successful thefts, the rates of all other observed behaviors were size dependent (Table 2). Small coho expended more effort than large coho in their foraging as seen in both their higher rates of strikes (Fig. 1a) and of theft attempts (Fig. 1d); the strike rate and capture rate both show stepwise decrease with increasing size of coho (Fig. 1a, b). The proportion of strikes that resulted in captures was on average 0.436 (95% CI 0.349–0.528) and did not differ significantly among coho of different sizes, indicating that small coho were just as adept at capturing prey as large coho.

**Table 2:** Results of generalized linear mixed-effects models comparing models with and without behavioral observations structured by size of the predator

Response variable	Model	*df*	NLL	*χ* ^2a^	${df}_{\chi^2}$ ^b^	*p* value
Rate of strike	Null	5	− 170.09			
	Size	7	− 94.36	151.45	2	< 0.001
Rate of capture	Null	5	− 115.99			
	Size	7	− 49.02	133.94	2	< 0.001
Rate of attempted thefts	Null	5	− 67.23			
	Size	7	− 61.19	12.09	2	0.002
Rate of successful thefts	Null	5	− 28.09			
	Size	7	− 28.06	0.07	2	0.967
Rate of being targeted by scroungers	Null	5	− 76.69			
	Size	7	− 50.40	52.57	2	< 0.001
Rate of prey escape	Null	5	− 65.78			
	Size	7	− 46.31	38.93	2	< 0.001
Proportion of strikes successful	Null	4	− 127.29			
	Size	6	− 126.82	0.93	2	0.628
Proportion of thefts successful	Null	4	− 37.72			
	Size	6	− 34.98	5.48	2	0.065

Rates were calculated as per coho per hour

*df* degrees of freedom of the model, *NLL* negative log likelihood

^a^Test statistic for the likelihood ratio test: *χ*^2^ =  − 2 ln(NLL_null_/NLL_size_)

^b^Degrees of freedom for the likelihood ratio test = *df*_size_ − *df*_null_

**Fig. 2:**
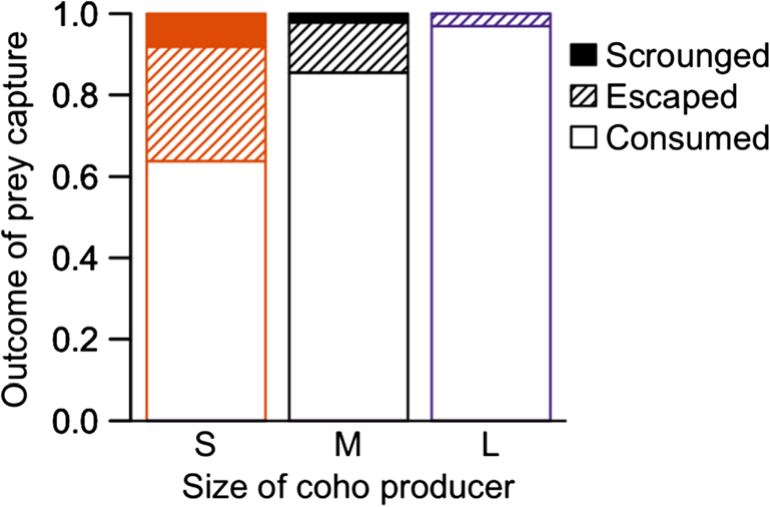
Proportional outcome for observations of prey captured by small coho (*n*_*obs*_ = 158), medium coho (*n*_*obs*_ = 187), and large coho (*n*_*obs*_ = 22)

However, small and medium coho had significantly lower proportions of theft attempts that were successful than large coho (Fig. 1h), as suggested by similar rates of successful thefts as large coho (Fig. 1e) despite more attempts (Fig. 1d) and a higher rate of losing prey to escapes (Fig. 1c). Small coho were most often targeted by scroungers, while large coho were least often targeted (Fig. 1f). These combined results lead to a dramatic difference in the outcome of captures for small, medium, and large coho (Fig. 2): small coho only retained 64% of their captures, losing the rest to escapes and theft, while large coho retained 95% of their captures and did not lose any prey to thefts.

The trials included groups of 10, 15, or 20 coho predators, and so, we also investigated whether predator group size improved any of the models for the observed behaviors. The rate of being targeted by scroungers increased with group size (Table S3), although the effect size was small in magnitude. The rates of attempted and successful thefts, however, did not increase significantly (Table S3). The proportion of strikes that were successful also tended to increase with group size, indicating perhaps more effective foraging ability in larger groups, though neither the rates of strike or capture were significantly affected by group size (Table S3).

## Producer-scrounger model

In this section, we develop a size-structured model of producer-scrounger dynamics based on our observations of coho salmon foraging. We begin with a basic model that describes the frequency-dependent payoffs to producers and scroungers in a foraging group and determine the Nash equilibrium at which an individual cannot increase its payoff by changing strategy. We then expand the model to incorporate two phenotypes in the foraging group that differ in their ability to scrounge and in their susceptibility to scrounging. We inform parameters in this phenotype-limited model using data from our empirical observations and determine the Nash equilibrium for each phenotype over a range of group sizes and parameter combinations.

### Basic model

We consider the per-capita payoff of producer and scrounger strategies as the average number of prey (or, more generally, food items) in the mouth of a producer (*P*) and the average number of prey in the mouth of a scrounger (*S*). The payoff is not necessarily equal to prey consumption, but is proportional to prey consumption for the basic model as we assume that the consumption rate is equal across the two strategies. The *P-S* dynamics are described by

1a $$ \frac{dP}{dt}=\lambda -\alpha qGP-\gamma P $$

1b $$ {\displaystyle \begin{array}{c}\ \\ {}\frac{dS}{dt}=\alpha \left(1-q\right) GP-\gamma S\end{array}}, $$

where *λ* is the per-capita rate a producer captures prey, *γ* is the combined rate of prey consumption and prey escape such that *γ*^−1^ is the mean handling time, *α* is the number of prey successful scrounged per scrounger per unit time, *q* is the proportion of scroungers in the group, and *G* is the number of individuals in the group (Table 3). The total rate of prey transfer from producers to scroungers is equal to α*qGP*(1 − *q*)*G*, where *qG* are the number of scroungers and (1 − *q*)*G* are the number of producers. The rate of prey transfer per producer and per scrounger differs between Eqs. (1a) and (1b) because we model the *average* payoff per producer or scrounger, and the number of producers in the group may differ from the number of scroungers. Note that scroungers can steal from scroungers as well as producers, but that transfer does not appear in this basic model because this does not change the average number of prey in the mouths of scroungers. Unlike previous models (e.g., Vickery et al. 1991; Barta and Giraldeau 1998, 2000), we do not assume that handling time is negligible but explicitly include a rate of prey consumption and escape (*γ*) that describes the susceptibility to scrounging; higher *γ* translates to faster handling times and therefore less opportunity for that prey to be stolen.

**Table 3:** Description of variables and parameters in the producer-scrounger model (Eqs. 1 and 7)

Description (units)	Symbol and assumed baseline value (range)					
	Basic model	Asymmetric model				
The average payoff to producers (prey)^a^	*P*	*P*_1_, *P*_2_				
The average payoff to scroungers (prey)^a^	*S*	*S*_1_, *S*_2_				
Proportion of predators that are scroungers^a^	*q*	*q*_1_, *q*_2_				
Rate of stealing (scrounger^−1^ h^−1^)	*α* = 0.40		*α*_11_	*α*_12_	*α*_21_	*α*_22_
		Scenario A	0.36	0.36	0.36	0.36
		Scenario B	0.36	0.12	0.60	0.36
		Scenario C	0.36	0.12	0.60	0.00
Rate of prey consumption and escape (h^−1^)^b,c^	*γ* = 1.2	*γ*_1_ = 1.002, *γ*_2_ = 1.914 (0.060 to 3.000)				
Rate of prey capture by producers (prey h^−1^)	*λ* = 0.60					
Group size (predators)^c^	*G* = 15 (0 to100)					
Proportion of predators that are small (unitless)^c^	–		θ = 0.5 (0 to 1)			

^a^Model variables

^b^Calculated from empirical observations for size-structured model; see “Parameterization” section

^c^A range of values were investigated in a sensitivity analysis

Many examples of producer-scrounger dynamics involve food resources that are divisible and assign a finder’s share to producers (e.g., Vickery et al. 1991; Ranta et al. 1996; Barta and Giraldeau 1998). We do not incorporate a finder’s share in our model, since our observations suggest that the prey in our system are not divided but consumed whole (see video in Supplementary Data). However, because we are modeling the average number of prey, the rates of prey capture and scrounging are not integer numbers. Decreasing the rate of scrounging in our model would effectively decrease the average number of prey in the mouths of scroungers and increase the average number of prey in the mouths of producers, and thus may have a similar effect as increasing the finder’s share in other *P-S* models.

For a given set of parameters, the dynamics of prey capture have an equilibrium where both *dP*/*dt* and *dS*/*dt* equal zero. This equilibrium payoff to producer and scrounger strategies is

2a $$ {P}^{\ast }(q)=\frac{\lambda }{\gamma +\alpha qG} $$

2b $$ {S}^{\ast }(q)=\frac{\left(1-q\right) G\alpha \lambda}{\gamma \left(\gamma +\alpha qG\right)} $$

which indicates that the equilibrium payoffs to producers and scroungers are both dependent on the proportion of scroungers, *q*.

We assume that the dynamics of behavioral changes between producer and scrounger strategies are based on the decisions of individuals aiming to maximize their prey intake. To model changes in the frequency of scrounging (*q*), we assume that the behavioral dynamics (i.e., the individual decisions to adopt a certain strategy) operate on a slower timescale, *τ*, than the dynamics of prey capture and consumption; that is, a given set of behavioral strategies rapidly results in an equilibrium of prey consumption rates for producers and scroungers (Eq. (2)), and we model how the frequency of scrounger and producer strategies changes in response to the payoffs given in Eq. (2):

3 $$ \frac{d q}{d\tau}=f\left({P}^{\ast },{S}^{\ast}\right). $$

We assume that an individual fish may switch its strategy after interacting with a fish who has a strategy with a superior payoff and that the probability of switching strategies is proportional to the increase in payoff that would be attained by switching. The law of mass action dictates that the rate of interaction between fish with different strategies is proportional to *q*(1 − *q*), and the increase in payoff equilibrium value derived from switching from producer to scrounger is S^*^(*q*) − P^*^(*q*). Therefore, assuming that the probability of interacting is independent of the increase in payoff equilibrium value, we multiply the two quantities together to get

4 $$ \frac{d q}{d\tau}\propto \left({S}^{\ast }(q)-{P}^{\ast }(q)\right)q\left(1-q\right) $$

Equation (4) describes an increase in the frequency of scrounging when the payoff to the scrounger strategy exceeds the payoff to the producer strategy, with the rate of change slowing as the proportion of scroungers approaches zero or one. The latter effect reflects that the rate of switching to the scrounger strategy (i.e., *dq*/*d*τ) depends on the proportion of predators available to make the switch as well as the number of predators demonstrating the more effective strategy. The rate of change in *q* is therefore zero at the boundaries *q =* 0 and *q* = 1.

The Nash equilibrium of this producer-scrounger game (i.e., the point at which an individuals’ payoff cannot be increased by changing strategy) is the frequency of scrounging in the group that corresponds to the stable equilibrium of Eq. (4), which occurs for the value of *q* at which P^*^(*q*) = S^*^(*q*):

5 $$ {q}^{\ast }=1-\frac{\gamma }{\alpha G}. $$

**Fig. 3:**
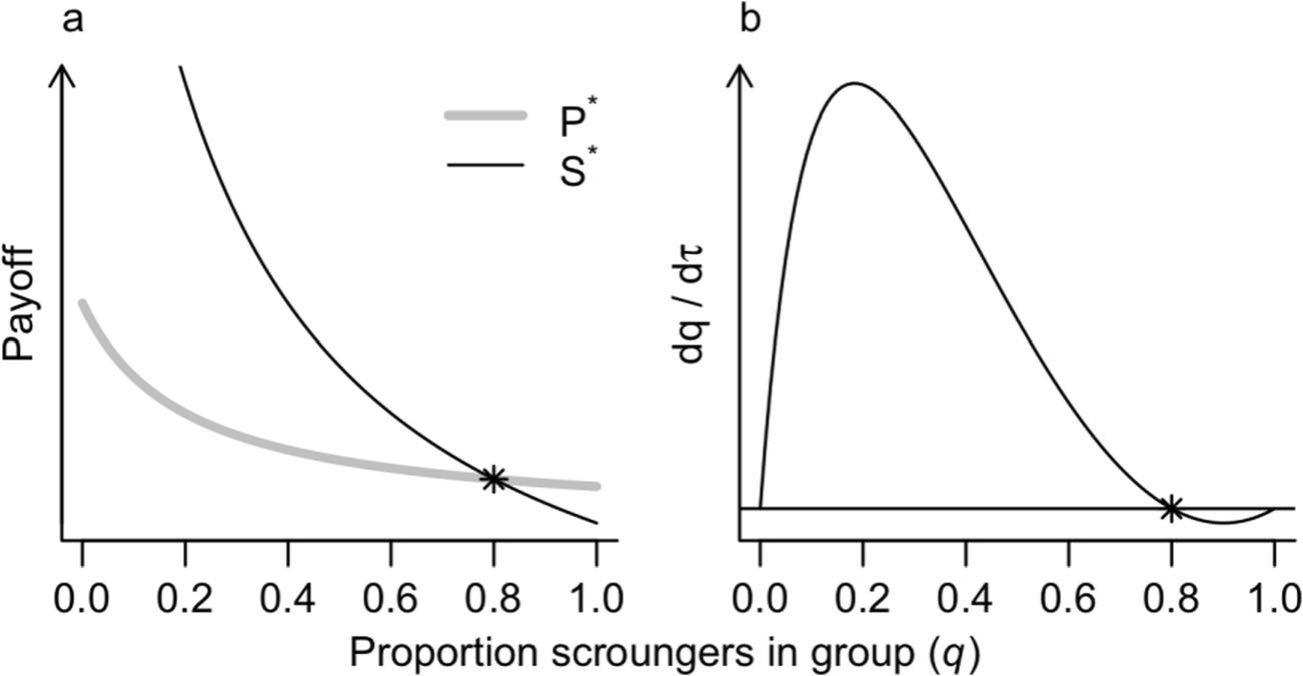
**a** Estimated payoff to producers (gray line) and scroungers (black line) in a group of 15 predators from the basic model as the proportion of scroungers in the group (*q*) increases from 0 to 1. The Nash equilibrium occurs where the lines cross and the payoff to the two strategies are equal (star). **b** Equation (4) describes the rate of change in the proportion of scroungers in the group, which is a function of the difference in payoff between producers and scroungers and the proportion of scroungers. In this case, the Nash equilibrium occurs where P^*^ = S^*^ at q^*^ = 0.8

For illustration, we investigated the payoff to producer and scrounger strategies as the proportion of scroungers in the group, *q*, increases from zero to one with parameter values chosen to be roughly consistent with the results of our observations (Fig. 1 and Table 3). See the section “Parameterization” below for further details. As *q* increases, the average payoff to both producers and scroungers declines as fewer producers make prey available to the group (Fig. 3a). The payoff to scroungers declines more steeply than the payoff to producers, resulting in the equal payoff to producers and scroungers at q^*^ = 0.8 (Fig. 3b). Note that Eq. (4) also has equilibria at q^*^ = 0 and q^*^ = 1, but these equilibria are not Nash equilibria because, for example, at q^*^ = 0, an individual may change its strategy to be a scrounger and receive a higher payoff.

From Eq. (5), the stable coexistence of the two strategies (i.e., q^*^ > 0) occurs when

6 $$ G>\frac{\gamma }{\alpha }, $$

which suggests that larger groups are more likely to have scroungers. Further, Eq. (5) suggests that if the predator group is of sufficient size that the scrounging strategy can invade, then the proportion of scroungers in the group increases with group size. This prediction is consistent with our findings from study 1, where the per-coho rate of theft attempts was higher in larger groups and with the findings of Vickery et al. (1991).

### Phenotype-limited model

Our empirical study indicated that large and small predators adopt different foraging strategies (Table 2) and that foraging strategies may depend on group composition (Online Supplement, Fig. S4b), suggesting that a predator’s optimal strategy may depend on its phenotype relative to the phenotypes of other members of its foraging group. Indeed, previous empirical studies have also suggested phenotype-dependent foraging strategies (e.g., Hansen 1986). In particular, we observed that large predators were more often successful in scrounging than small predators and that small predators were most often the target of scrounging. To investigate the influence of predator size on producer-scrounger dynamics, we modified Eq. (1) to differentiate the payoff to two phenotypes, which we call small and large, and account for theft between small and large scroungers. The equations describing the dynamics of food acquisition by small (subscript 1) and large (subscript 2) producers (*P*) and scroungers (*S*) are7a7b7c7d

Equations (7a)–(7d) contain a new parameter, θ, which is the proportion of predators in the group that are small. The rates of stealing are size-structured such that there are four separate parameters denoted α_*ij*_ describing the rates of stealing by size *i* on size *j*. The combined rates of consumption and escape are also size-structured (*γ*_1_ and *γ*_2_ for large and small predators, respectively), and the proportion of scroungers may differ for small and large predators (*q*_1_ and *q*_2_ for large and small predators, respectively; Table 3). The per-capita rate at which producers capture prey, *λ*_1_ and *λ*_2_, may also differ between small and large predators, but for our initial exploration of the model, we assumed that *λ*_1_ = *λ*_2_ = *λ*. Although small and large predators had different capture rates in our empirical observations (Fig. 1), we assume that this was due to different proportions of producers in the size classes.

We calculated the equilibrium payoff to small producers, large producers, small scroungers, and large scroungers from Eqs. (7a) to (7d), respectively. As in the analysis of the basic model, we assume that the dynamics of behavioral shifts between producer and scrounger strategies operate on a slower timescale (*τ*) than the dynamics of prey capture and consumption. Further, we assumed matching logistic functions describing the rates of change in the proportion of small and large predators that are scroungers:

8a $$ \frac{d{q}_1}{d\tau}=\left({S}_1^{\ast}\left({q}_1,{q}_2\right)-{P}_1^{\ast}\left({q}_1,{q}_2\right)\right){q}_1\left(1-{q}_1\right) $$

8b $$ \frac{d{q}_2}{d\tau}=\left({S}_2^{\ast}\left({q}_1,{q}_2\right)-{P}_2^{\ast}\left({q}_1,{q}_2\right)\right){q}_2\left(1-{q}_2\right), $$

where *P*_1_^*^, *P*_2_^*^, *S*_1_^*^, and *S*_2_^*^ are the equilibrium payoffs for small producers, large producers, small scroungers, and large scroungers, respectively, and *q*_1_ and *q*_2_ *ϵ* [0,1].

The equations for the equilibrium payoffs of the different strategies were much more complex than those in the basic model, so rather than analytically describing the conditions under which both producer and scrounger strategies would coexist, we analyzed Eq. (7) numerically, with parameter values informed by our empirical observations (see the following section).

#### Parameterization

Parameters and their assumed baseline values (i.e., parameters not being varied in a sensitivity analysis) are summarized in Table 3. The following is a more detailed justification of our parameterization. In the equations describing payoffs to the different strategies (*P*_1_^*^, *P*_2_^*^, *S*_1_^*^, and *S*_2_^*^; see Online Supplement), *λ* scales each of the payoffs, affecting the absolute—but not relative—predicted results. We set *λ* = 0.6 h^−1^, which gave us payoffs that were biologically reasonable (i.e., *P*_1_^*^, *P*_2_^*^, *S*_1_^*^, and *S*_2_^*^ < 1, since most predators are unlikely to have more than one prey in their mouth at any given point in time). This value is also in the order of magnitude of our empirical observations of the capture rate of producers (i.e., (1 − *q*) *λ* from 0.1 to 3.2 h^−1^; Fig. 1b).

In our additional observations (see Online Supplement), we found that large predators consumed prey more quickly than small predators. To determine the combined rate of prey consumption and escape (*γ*), we used a survival analysis of the observed time to prey consumption or escape for both small and large predators. This yielded estimates of *γ*_1_ = 1.0 h^−1^ and *γ*_2_ = 1.9 h^−1^. We also investigated the sensitivity of the equilibrium proportion of scroungers to the combined rate of consumption and loss for *γ*_1_ and *γ*_2_ in the range 0.06 to 3.00 h^−1^, in increments of 0.06 h^−1^.

For the basic model, we assumed that the rate of stealing (i.e., successful thefts) was *α* = 0.4 scrounger^−1^ h^−1^. This value is an order of magnitude higher than our empirical observations for rates of successful thefts because the latter inherently includes the proportion of the group that was scrounging (i.e., the rate of successful thefts in Fig. 1e relates to *qα* in the model, not *α*). For the size-structured model, the relative stealing rates among size classes were of more interest than the absolute rates because we were interested in the qualitative producer-scrounger dynamics, and we did not attempt to make quantitative predictions for a given group size. We examined three different scenarios for the relative rates of stealing among size classes. First, for scenario A, we assumed that rates of stealing were equal among the size classes. The other two scenarios (B and C) were based on our empirical observations and were constrained such that large predators stole from small predators at the highest rate and small predators stole from large predators at the lowest rate (Table 3). For scenario B, we assumed that large predators stole from large predators at the same rate that small predators stole from small predators. The data showed that large predators stealing from large predators were rare, so for scenario C, we assumed that *α*_22_ = 0 scrounger^−1^ h^−1^ and large predators stole only from small predators.

We investigated the dynamics over a range of group sizes from *G* = 1 to 100. As a baseline value, we assumed that the proportion of predators that were small was θ = 0.5. We varied this parameter from 0 to 1 in subsequent simulations to observe how it affected the payoff to small and large predators because additional empirical observations suggested that the size of the predator group may affect behavioral dynamics (Online Supplement).

#### Numerical method

**Fig. 4:**
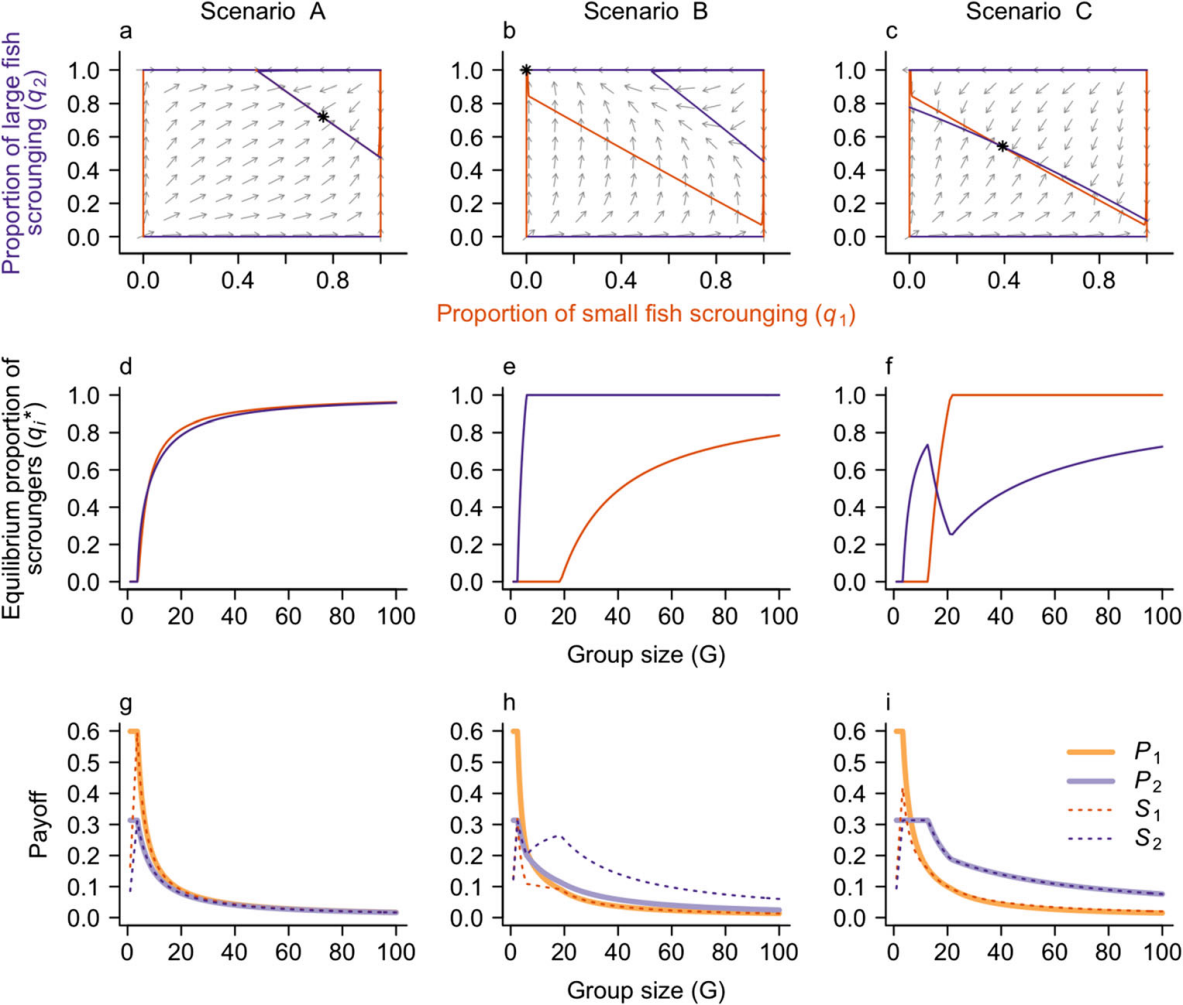
Results from scenarios A–C capturing three different relative rates of stealing among size classes. **a**–**c** The phase plane for the dynamics of *q*_1_ (red, *x*-axis) and *q*_2_ (blue, *y*-axis) for a predator group size of *G* = 15, including nullclines where *dq*_1_/*d*τ = 0 (red lines) and *dq*_1_/*d*τ = 0 (blue lines). Gray arrows show the direction of flow, and the star indicates the Nash equilibrium. **d**–**f** The Nash equilibrium proportion of small predators (red) and large predators (blue) that are scroungers over increasing group size. The vertical dashed line indicates *G* = 15, corresponding to the phase planes in **a**, **d**, and **g**. **g**–**i** The equilibrium payoff to each strategy (when the proportion of scroungers is allowed to assume the Nash equilibrium in **d**–**f**) over increasing group size

We used the package phase R (Grayling 2014) to plot the *q*_1_-*q*_2_ phase plane and determine the equilibrium proportion of scroungers of each size class. Nullclines of Eq. (7) (i.e., the curves corresponding to *dq*_*1*_/*d*τ = 0 and *dq*_2_*/d*τ = 0) represented regions of the strategy space for which one phenotype could not gain additional benefit by switching strategy. Where *q*_1_ and *q*_2_ nullclines intersect, scrounging proportions are at equilibrium (*q*_1_^*^, *q*_2_^*^). Dynamically stable equilibria represented the Nash equilibrium for small and large predators. These equilibria were found using the steady() function in the R package rootSolve (Soetaert 2009) and were confirmed by examining the flow field of the *q*_1_-*q*_2_ phase plane (Fig. 4a–c).

#### Model results

The producer-scrounger dynamics in the phenotype-limited model depended on the relative rates of stealing among phenotypes (Fig. 4). When the rates of stealing were independent of phenotype (scenario A), the producer-scrounger dynamics were similar for small and large predators (Fig. 4d) although there were slight differences in the proportion of scroungers between phenotypes; there was a pattern of more scrounging in large predators at small group sizes and more scrounging among small predators in large groups (Fig. 4d). This difference can be understood because although the rates of stealing were equal between phenotypes, the equilibrium payoffs for the asymmetric model also depended on the combined rate of consumption and loss, which was smaller for small predators.

When large predators stole indiscriminately from large and small individuals at a higher rate than small predators stole from either size class (scenario B), the model predicted a higher proportion of scroungers for large than for small predators (Fig. 4e). Further, all large predators tended to scrounge (i.e., *q*_2_^*^ = 1) before the scrounger strategy was able to invade for small predators. There was a minimum group size below which no small predators would adopt a scrounging strategy (Fig. 4e).

When large scroungers were limited to stealing only from small predators (scenario C), producer and scrounger strategies coexisted for both small and large predators at moderate group sizes (Fig. 4c, f). In contrast to scenario B, as group size increased, all small predators tended to scrounge (*q*_1_^*^ = 1) while some large predators acted as producers (*q*_2_^*^ < 1). This can be understood because the success of the scrounging strategy was limited for large predators when *α*_22_ = 0. Indeed, when we increased the proportion of the group that was small, there was a corresponding increase in the proportion of large predators adopting the scrounging strategy (Fig. 6f).

In all scenarios, the payoff to both large and small producers declined with increasing group size as the scrounging strategy became more prevalent (Fig. 4g–i). In small groups (*G* < 10), small producers had the highest payoff, but this quickly changed as group size increased and large scroungers became more prevalent. Large scroungers generally had the highest payoff, except when all stealing rates were equal (scenario A). The changes in payoff with group size (Fig. 4g–i) suggest that large predators, especially large scroungers, have higher payoff in large groups relative to small predators, and small predators have higher payoff in small groups.

The average payoff to the group, calculated as the payoff to each strategy multiplied by the proportion of the group adopting that strategy at behavioral equilibrium, decreased monotonically with group size. The equations for equilibrium payoff indicate that this decrease in payoff with group size is due to the social parasitism of scroungers and not simply due to a dilution of resources among more individuals. In the absence of scroungers (i.e., *q*_1_ = *q*_2_ = 0), the equilibrium payoffs to producers are *P*_1_^*^ = *λ* / *γ*_1_ and *P*_2_^*^ = *λ* / *γ*_2_, which are independent of group size. The model therefore predicts that producers acquire the same amount of resources regardless of how many predators are in the group. However, when the proportions of scroungers are allowed to go to the Nash equilibrium (i.e., *q*_1_ = *q*_1_^*^ and *q*_2_ = *q*_2_^*^), the payoff to producers is a decreasing function of the proportion of scroungers (Fig. 4g–i).

**Fig. 5:**
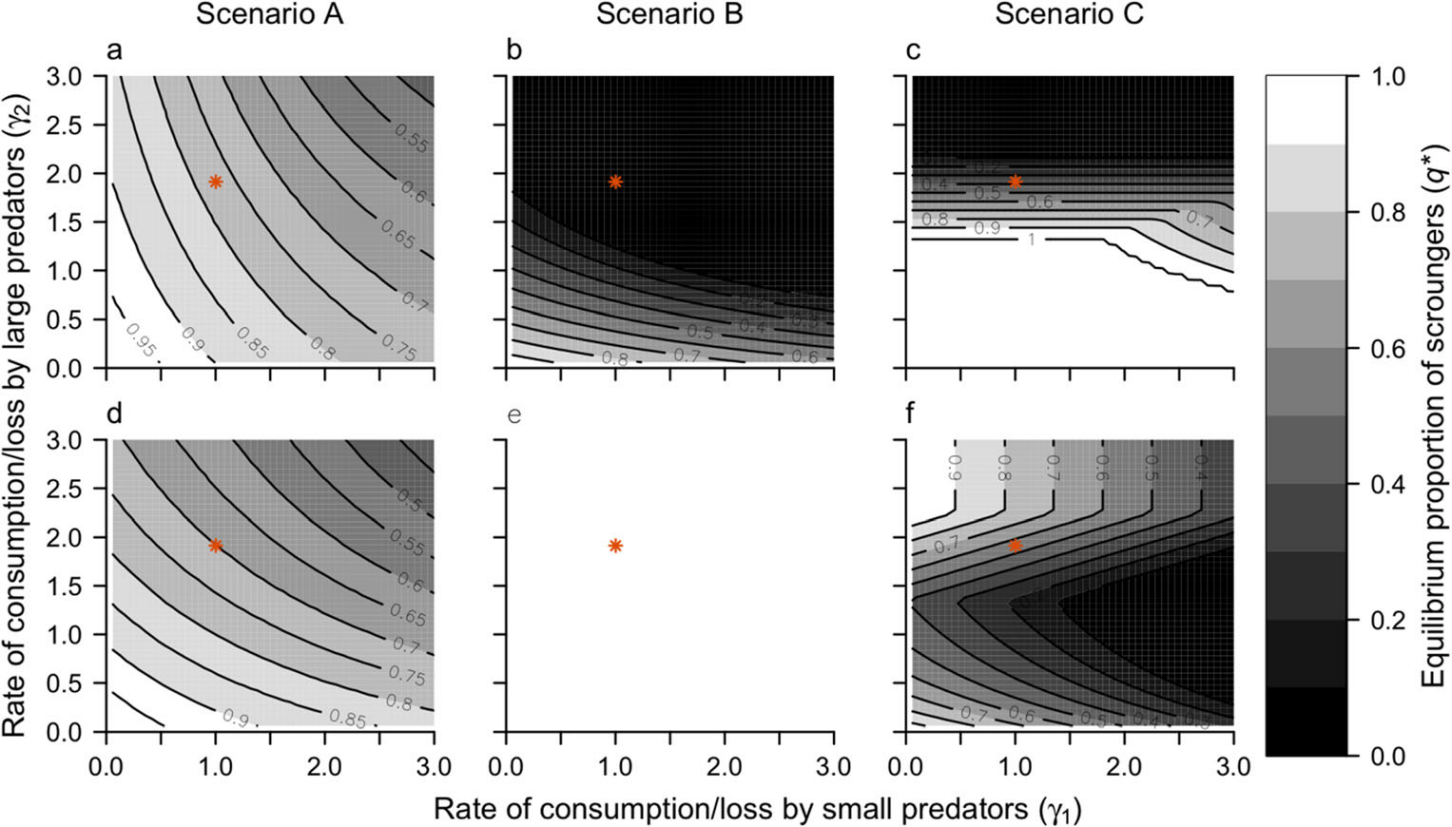
The equilibrium proportion of scroungers for small predators (*q*_1_^*^, **a**–**c**) and large predators (*q*_2_^*^, **d**–**f**) as a function of *γ*_1_ (*x*-axis) and *γ*_2_ (*y*-axis). Each column corresponds to one of the scenarios A–C for the relative rates of stealing among size classes. The red point indicates the values of *γ*_1_ and *γ*_2_ derived from empirical observations (Table 3)

In general, the equilibrium proportion of scroungers was inversely related to the combined rates of consumption and loss, *γ*_1_ and *γ*_2_ (Fig. 5a–d), as high rates of consumption/loss meant faster handling times and fewer opportunities for scrounging. In scenario A, for which rates of stealing were equal between phenotypes, the simple pattern scrounging increasing with decreasing combined rates of consumption and loss was evident for small and large predators (Fig. 5a, d). For scenario B, all large predators tended to scrounge regardless of changes to *γ*_1_ and *γ*_2_ (Fig. 5e). Conversely, small predators tended to produce, except when *γ*_1_ and *γ*_2_ were small (Fig. 5b). In scenario C, the equilibrium proportion of small predators that scrounged increased with decreasing *γ*_2_, but was relatively unaffected by changes to *γ*_1_. When small predators were all producers (i.e., *γ*_2_ > 2.4 h^−1^; Fig. 5c), the equilibrium proportion of large predators that were scroungers increased with decreasing *γ*_1_ but was independent of *γ*_2_ because large scroungers only stole from small predators in scenario C. As *γ*_2_ decreased from 2.4 to 1.2 h^−1^ and the proportion of small predators scrounging increased from 0 to 1, the proportion of large predators scrounging decreased. Below *γ*_2_ ≈ 1.2 h^−1^, all small fish were scroungers (Fig. 5c), and as *γ*_2_ declined, *q*_2_^*^ increased (Fig. 5f).

**Fig. 6:**
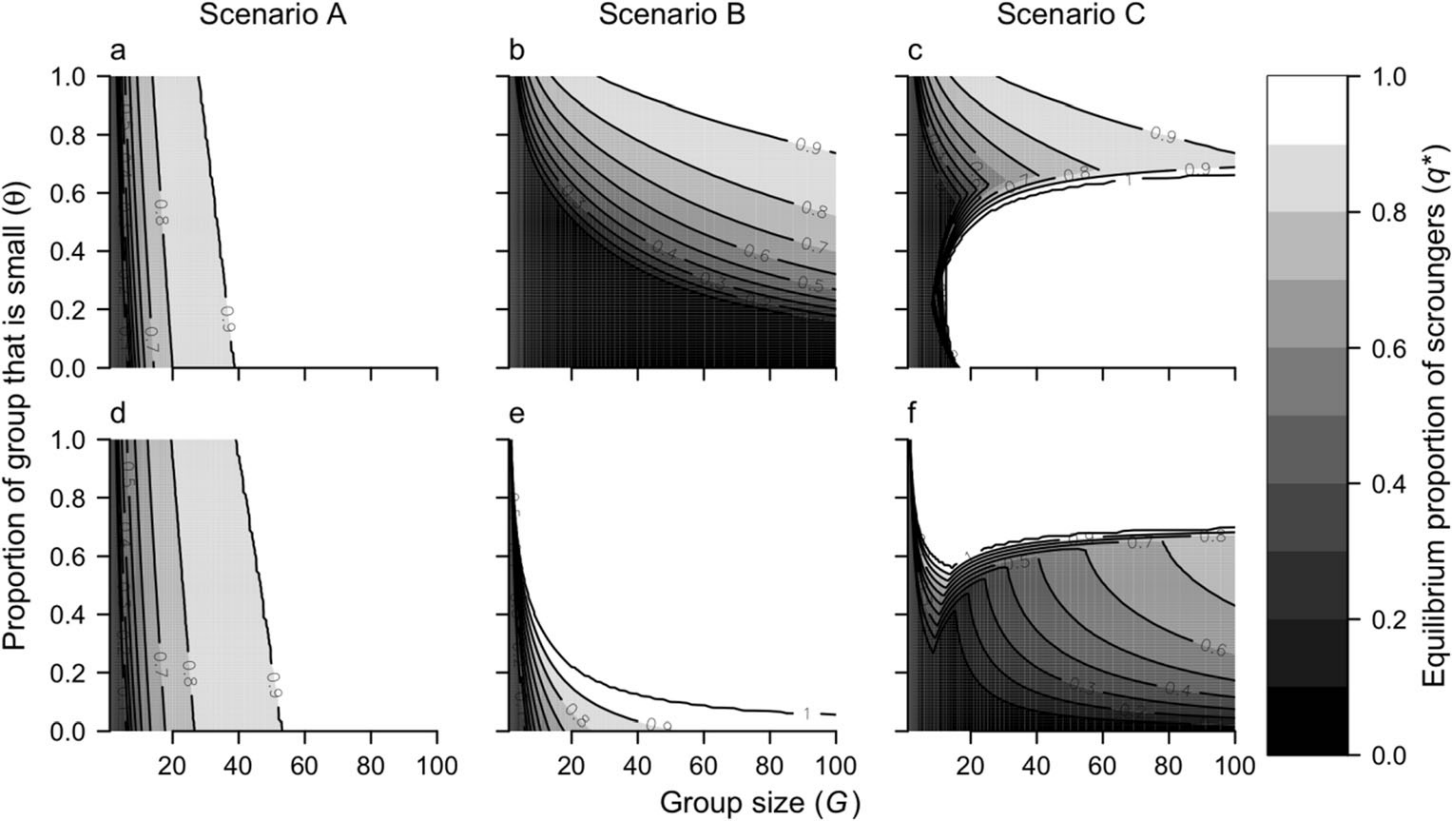
The equilibrium proportion of scroungers for small predators (*q*_1_^*^, **a**–**c**) and large predators (*q*_2_^*^, **d**–**f**) as a function of group size, *G* (*x*-axis), and the proportion of the group that is small, θ (*y*-axis). Each column corresponds to one of the scenarios A–C for the relative rates of stealing among size classes

We investigated the sensitivity of producer-scrounger dynamics to the proportion of predators that were small because our additional empirical observations suggested that scrounging was more prevalent in groups of mixed-sized predators (Online Supplement). In scenario A, the rates of stealing were independent of predator size, and so, the proportion small in the group had little effect on the equilibrium proportion of scroungers for small or large predators, although there was a strong trend of increasing scrounging with group size (Fig. 6a, d). In scenario B, the equilibrium proportion of small predators that were scroungers (*q*_1_^*^) increased with group size and with the proportion of predators that were small (Fig. 6b). A similar pattern arose for the equilibrium proportion of large predators that were scroungers (*q*_2_^*^; Fig. 6e), but the proportion of scroungers was much higher for large predators than for small predators. In scenario C, the proportion of small predators that were scroungers changed nonlinearly as the proportion of that group that was small increased, but was generally lowest for mixed groups (θ = 0.7; Fig. 6c). As θ approached one and groups were mainly comprised of small predators, scrounging was prevalent for both small and large predators (provided group size was large enough; Fig. 6c, f). However, in groups of only large predators (θ = 0), no large predators scrounged (Fig. 6f) as large predators could only scrounge from small predators in scenario C. At a group size of *G* ≈ 10, small predators began to scrounge (Fig. 4f) which changed the equilibrium dynamics of *q* for large predators, resulting in a sudden decline in *q*_2_^*^ as *G* increased further for a given θ (Fig. 6f). The proportion of scroungers in the entire group, irrespective of size (i.e., θ*q*_1_^*^ + (1 − θ)*q*_2_^*^), increased with group size and proportion small, similar to scenario B. Contrary to our empirical observations, none of the scenarios showed the highest rates of scrounging in groups with mixed phenotypes.

## Discussion

In social foraging groups, individuals make quick decisions about whether to forage for themselves or steal prey from conspecifics, but they may not be able change other traits that influence their foraging success on such short timescales. These other traits that are not so easily changed, such as body size, may influence their ability to steal prey and thus dictate their optimal foraging strategy. As our results show, a behavioral stable strategy (BSS) may therefore emerge where different physical phenotypes may correspond to different optimal foraging strategies. Empirical evidence from previous studies seems to suggest that larger or more dominant individuals tend to scrounge (Hansen 1986). Many studies found that dominant individuals exploit food found by subordinates (Baker et al. 1981; Rohwer and Ewald 1981; Czikeli 1983; Theimer 1987; Caraco et al. 1989; Wiley 1991; Stahl et al. 2001; Held et al. 2002; Liker and Barta 2002), though some studies found no effect of dominance on the frequency of scrounging behavior (Giraldeau and Lefebvre 1986; Beauchamp 2000, 2006). There is also some empirical evidence that inefficient foragers, such as individuals that are young or inexperienced, use the scrounging strategy more often (Steele and Hockey 1995; Bautista et al. 1998; Goss-Custard et al. 1998; Beauchamp 2000, 2006).

Our observational study of foraging behavior in juvenile coho salmon showed that large individuals were more successful at scrounging and took less time to handle prey, while small individuals tended to capture more prey, acting as producers, but were more susceptible to scrounging as they took longer to handle prey. Although the majority of foraging behavior tended to occur near the surface, we may have underestimated rates of prey escape because predators tended to move to deeper waters once they had captured or stolen prey, perhaps to avoid scrounging from conspecifics and predation by birds. Grand (1997) found that juvenile coho salmon can distinguish the relative competitive abilities of conspecifics and choose foraging locations in streams accordingly. Our results indicate that in marine settings, where juvenile coho form foraging groups in the water column that feed on smaller fish and invertebrates, coho also assess conspecifics when choosing producer or scrounger strategies, such that smaller coho are primarily producers and are also targeted by scroungers more often than large coho.

In their review, Giraldeau and Beauchamp (1999) state that when phenotypes are discrete (e.g., sex), one phenotype will act exclusively as producers while the other act exclusively as scroungers; however, for continuous phenotypes (e.g., age, body size, dominance), there may be an equilibrium in which different phenotypes play both strategies depending on a variety of situation-specific variables. Here, we treated a continuous phenotype (i.e., size) as discrete (i.e., large and small), and yet found conditions under which both sizes will employ both strategies, but at different frequencies. This result expands the set of outcomes that can arise for group foraging behavior in phenotype-limited asymmetric games.

In general, our model predicted that as group size increased, so would the Nash equilibrium frequency of scroungers in the group. We assumed complete incompatibility between producer and scrounger strategies (Barnard 1984; Giraldeau and Caraco 2000), such that an individual cannot simultaneously be a producer and a scrounger. Thus, as the frequency of scroungers increased, the number of producers declined and the total number of prey captured by the group decreased. This implies a mechanism by which kleptoparasitism may regulate upper limits on group sizes due to declining per-capita food availability arising from increased scrounging frequencies in larger groups. However, group sizes will also be affected from a trade-off between group-size benefits, such as predator avoidance (which may regulate lower limits on group size), and group-size costs, such as kleptoparasitism. For archerfish, the likelihood of kleptoparasitism increases as group size increases from three to five fish, after which it plateaues (Dill and Davis 2012). However, although there is a higher rate of scrounging in groups with more members, the benefits of increased group size may outweigh the cost of losing more prey to scroungers in some cases (Ranta et al. 1996). For example, both goldfish and minnows locate food items more quickly as group size increases (Pitcher et al. 1982). The coho predators in our study are themselves subject to predation by birds and other predators, and group formation may make individual coho less susceptible to predation (e.g., via the confusion effect; Landeau and Terborgh 1986). Coho may also rely on multiple attacks from a group of predators to split and confuse schools of prey, making it easier to capture individual prey. We did not observe coordinated herding by coho predators, but in preliminary trials, coho would not attack a group of prey when they were alone or with only one or two other predators, suggesting that the size of coho groups somehow facilitates predation.

Group composition—the relative abundance of different phenotypes—also affected producer-scrounger dynamics in our model. In general, the proportion of scroungers was highest for large groups comprised mainly of small phenotypes. Previous studies have suggested that producer-scrounger strategies will be plastic in response to group composition. For example, Morand-Ferron et al. (2011) found that nutmeg mannikins (*Lonchura punctulata*) adjusted their foraging strategy when group composition was changed: when individuals that used the scrounging strategy most were placed into the same flock, the frequency of producing and scrounging in the flock was no different from that of flocks composed entirely of individuals which formerly used the scrounging strategy least. However, when groups were re-assorted, there was a lag period of ~ 3 days before producer-scrounger dynamics equilibrated, presumably due to the time it took individuals to assess the relative payoffs of the different strategies. This lag might explain why our empirical observation that scrounging was more prevalent in mixed size groups (Online Supplement, Fig. S4), differed from model predictions, which suggested that scrounging increased with the proportion of the group that was small (Fig. 6). Our model assumed that the behavioral dynamics were at equilibrium, but in our observational study, coho were not given the opportunity to assess relative payoffs in their group prior to observations being made. Thus, the producer-scrounger dynamics in the size-assorted study (Online Supplement) may have been influenced by previous group composition. This was less likely to have influenced results of our initial observations where groups were haphazardly selected from the population and the proportion of large and small phenotypes did not change significantly from the group composition in the holding pen.

For the phenotype-limited game, the payoff to large scroungers was generally the highest, which would suggest that individuals would be best to join groups where they are the largest individual or attempt to evict individuals larger than themselves from their group (as has been reported for northern harriers; Temeles 1990). However, in nature, schools of fish appear well size-assorted (Hoare et al. 2000). It may be that a balance of social foraging, which favors the largest in a group, and predation risk, which increases for individuals that stand out (Landeau and Terborgh 1986), determine size-assorting in social groups (Ranta et al. 1993). In our observational study and in our model, we did not allow for individuals to enter or leave groups, but this would be an interesting avenue for future study.

Our model advances the theory of asymmetric games of producers and scroungers by giving the conditions under which producer *and* scrounger strategies might coexist in two different phenotypes. Ranta et al. (1996) were the first to consider intraspecific heterogeneity in foraging traits, but in their model, individuals could simultaneously act as producers and scroungers (i.e., information-sharing or complete compatibility between strategies). An information-sharing model may be appropriate for some fish species, but for coho, our observations suggest that predators pursuing a conspecific (i.e., scrounging) cannot simultaneously pursue a prey item and vise versa. Further, we observed that some (larger) individuals predominantly scrounged and some (smaller) individuals predominantly acted as producers, rarely joining in other’s catch. These observations suggest that a producer-scrounger model is appropriate for our system, and others have argued that producer-scrounger models may be more useful for studying group foraging in general (Beauchamp and Giraldeau 1996).

Barta and Giraldeau (1998) analyzed an alternative-option producer-scrounger game by including dominance rank as a continuous trait affecting competitive ability of scroungers, and found that there was a threshold in dominance above which all individuals will play one strategy and below which all individuals will play the other strategy. Our results, based on two discrete phenotypes, reinforced their findings in that one phenotype (i.e., large) tended to play the scrounger strategy while the other phenotype played producer. Broom et al. (2008) considered four phenotypes based on attack and retaliation tendencies: hawk, dove, retaliator, and marauder. These four strategies are similar to our large scrounger, small producer, large producer, and small scrounger strategies, respectively, but we explored a wider range of behavior by continuously varying the rates of scrounging (*α*_*ij*_) that controlled scrounging success for the two different phenotypes.

Depending on the metabolic or other costs to different strategies and phenotypes, which we did not explicitly consider, producer-scrounger dynamics may result in divergent or convergent growth trajectories for small and large individuals. Reinhardt (1999) found that in the absence of a predation threat, juvenile coho that were larger and more aggressive grew faster, which would suggest divergent growth trajectories for small and large individuals. This is further supported by our model prediction that scrounging generates higher payoffs for larger foragers. Our empirical observations showed that smaller coho took much longer to consume prey, were more likely to have prey escape, and were more vulnerable to scrounging. Taken together, these results suggest that producer-scrounger dynamics may benefit relatively large fish while suppressing growth of small fish (although this may not be the case if foraging success, *λ*, differs among phenotypes—a case we did not consider here). For coho predators, growth in early life is thought to be a key determinant of fitness: analysis of scale growth rings indicates that the fastest-growing juveniles are most likely to survive to recruitment and spawning (Beamish and Mahnken 2001). Producer-scrounger dynamics may, therefore, lead to self-reinforcing effects on foraging success, body growth, and ultimately fitness. Much more work is required to elucidate these relationships.

Producer-scrounger games in juvenile coho salmon may have implications for their growth and survival, but the model we have presented can be applied broadly. Variation in body size among individuals is common across species, and such variation often influences the probability of success in acquiring food (e.g., Hansen 1986), mates (e.g., Shine et al. 2000), and habitat (e.g., Gherardi 2006). More generally, it is common to observe heterogeneity in many traits associated with foraging success and social dominance. Incorporating size-structure or trait-mediated effects into models of game-theoretic behavioral dynamics is an important step in understanding animal behavior, and the effects of behavior on growth, survival, and fitness.

## Data accessibility

Data and R code reproducing the analyses are freely available at https://github.com/sjpeacock/Producer-Scrounger.

## Electronic supplementary material

ESM 1 (DOCX 787 kb)
