# Supplementary material for: An asymmetric producer-scrounger game: body size and the social foraging behavior of coho salmon
Source: Theor Ecol. 2018 May 1;11(4):417–31. doi: 10.1007/s12080-018-0375-2 (PMC6405016; doi:10.1007/s12080-018-0375-2)
Supplement: Supplementary file 2 — (DOCX 787 kb) [file 12080_2018_375_MOESM1_ESM.docx]

# Additional Figures and Results

Table S1. Results of generalized linear mixed-effects models comparing the model including size with a more complex model that includes the interaction between prey infestation with sea lice and size of predator. Rates are per coho per hour. The rate of prey escape was the only variable significantly affected by lice, but the size-structure did not change in this case; the rate of escapes from small predators was even higher with infested prey (Fig. S2).

| Response variable | Predictor variable(s) | *df* * | NLL† | $\chi^{2}$‡ | ${df}_{\chi^{2}}$§ | *p* |
| --- | --- | --- | --- | --- | --- | --- |
| Rate of strike | size | 7 | -94.36 |  |  |  |
|  | size*lice | 10 | -92.15 | 4.42 | 3 | 0.220 |
| Rate of capture | size | 7 | -49.02 |  |  |  |
|  | size*lice | 10 | -47.17 | 3.69 | 3 | 0.297 |
| Rate of attempted thefts | size | 7 | -50.40 |  |  |  |
|  | size*lice | 10 | -48.54 | 3.73 | 3 | 0.292 |
| Rate of successful thefts | size | 6 | -126.82 |  |  |  |
|  | size*lice | 9 | -126.18 | 1.28 | 3 | 0.733 |
| Rate of being targeted by scroungers | size | 7 | -61.19 |  |  |  |
|  | size*lice | 10 | -60.43 | 1.51 | 3 | 0.679 |
| **Rate of prey escape** | **size** | **7** | **-46.31** |  |  |  |
|  | **size*lice** | **10** | **-39.07** | **14.48** | **3** | **0.002** |
| Proportion of strikes successful | size | 7 | -28.06 |  |  |  |
|  | size*lice | 10 | -26.47 | 3.17 | 3 | 0.367 |
| Proportion of thefts successful | size | 6 | -34.98 |  |  |  |
|  | size*lice | 9 | -33.87 | 2.22 | 3 | 0.528 |

Notes: **df*: degrees of freedom of the model. † NLL: negative log likelihood. ‡ Test statistic for the Likelihood Ratio Test: $\chi^{2}=-2\ln\left( \mathrm{NL}L_{\mathrm{null}}/NLL_{\mathrm{size}} \right)$. § degrees of freedom for the Likelihood Ratio Test = *df*_size_ - *df*_null_.


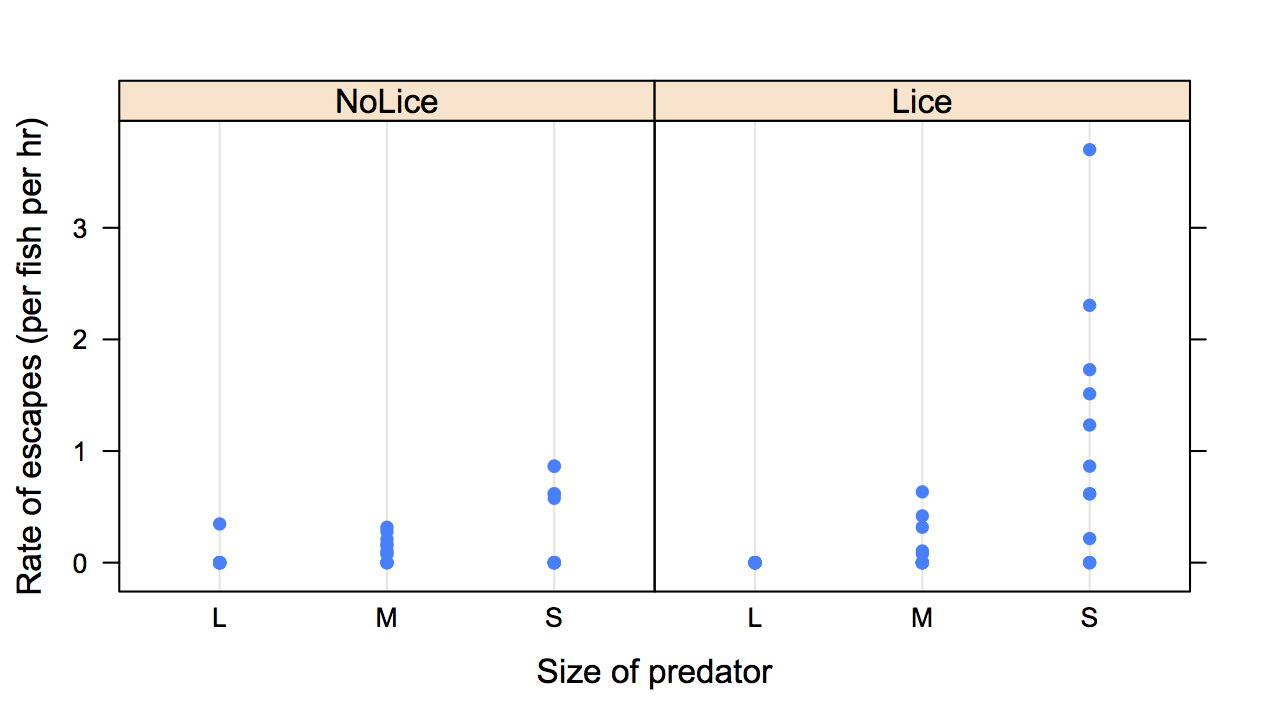


Figure S 1. The rate of prey escape (per fish per hour) structured by size of the predator and trials with and without infested prey. Each point represents an independent trial (total of 24 trials). There was a significant interaction between the size of predator and infestation (Table S2), however this was not due to a reordering of the size effects but rather because the rate of prey escapes was much higher from small predators with infested prey.

Table S2. Comparison of the size of coho salmon (fork length, mm) selected for the study vs. those that were not selected (i.e., remaining population in holding pen). The mean fork length of selected and remaining coho are given, as well as the results of independent two-sample two-sided Kolmogorov-Smirnov tests comparing the distributions of selected and remaining coho. In all trials, the size distribution of selected coho did not differ significantly from the size distribution of those that remained in the holding pen (*p*>0.05).

| **Year** | **Location** | **Expt** | **Forklength (mm)** | | | |
| --- | --- | --- | --- | --- | --- | --- |
|  |  |  | **Selected** | **Remaining** | ***D**** | ***p*** |
| 2013 | Kingcome | 6B | 110.3 | 116.4 | 0.17 | 0.731 |
|  |  | 6A | 108.5 | 116.8 | 0.22 | 0.381 |
|  | Bond | 10B | 121.3 | 119.7 | 0.24 | 0.308 |
|  |  | 10A | 117.0 | 120.5 | 0.18 | 0.641 |
|  |  | 11A | 116.3 | 120.6 | 0.22 | 0.382 |
|  |  | 12B | 121.3 | 119.7 | 0.08 | 1.000 |
|  |  | 12A | 122.0 | 119.5 | 0.20 | 0.497 |
|  |  | 13A | 122.5 | 119.3 | 0.13 | 0.801 |
| 2014 | Bond | 13B | 121.0 | 121.6 | 0.10 | 1.000 |
|  |  | 14B | 115.0 | 122.2 | 0.24 | 0.668 |
|  |  | 14A | 121.0 | 121.6 | 0.21 | 0.804 |

* Test statistic for the Kolmogorov-Smirnov test.


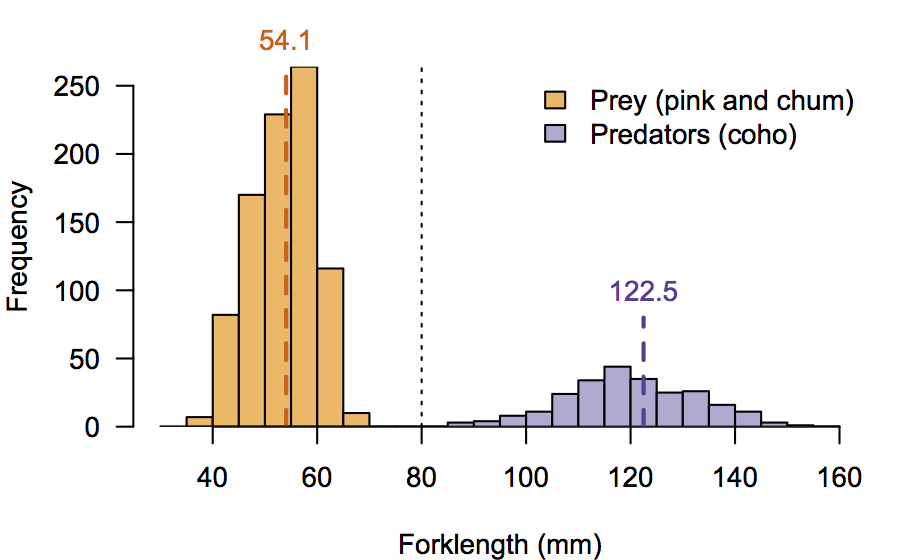


Figure S 2. The size distributions of all pink and chum salmon prey (orange) and coho salmon predators (purple) used in the study). The vertical dashed lines indicate the mean fork length of prey and predators, and the vertical dotted line indicates the threshold size below which predators were excluded from experiments (80 mm).

Table S3. Results of generalized linear mixed-effects models comparing the best model from Table 2 (main text) with a more complex model that included the total number of coho in the trial (10, 15, or 20) as a continuous predictor variable. Rates are per coho per hour. The rate of being targeted by scroungers and proportion of strikes that were successful increased significantly with the total number of coho in the trial (bold).

| Response variable | Predictor variable(s) | *df* * | NLL† | $\chi^{2}$‡ | ${df}_{\chi^{2}}$§ | *p* | Slope of effect^‖^ |
| --- | --- | --- | --- | --- | --- | --- | --- |
| Rate of strike | size | 7 | -94.36 |  |  |  |  |
|  | size+tot.coho | 8 | -93.21 | 2.30 | 1 | 0.129 | -0.04 (-0.09, 0.00) |
| Rate of capture | size | 7 | -49.02 |  |  |  |  |
|  | size+tot.coho | 8 | -49.02 | 0.00 | 1 | 0.962 | 0.00 (-0.05, 0.05) |
| Rate of attempted thefts | size | 7 | -61.19 |  |  |  |  |
|  | size+tot.coho | 8 | -59.48 | 3.41 | 1 | 0.065 | 0.10 (0.00, 0.19) |
| Rate of successful thefts | - | 5 | -28.06 |  |  |  |  |
|  | tot.coho | 6 | -26.33 | 3.51 | 1 | 0.061 | 0.18 (-0.02, 0.38) |
| **Rate of being targeted by scroungers** | size | 7 | -50.40 |  |  |  |  |
|  | **size+tot.coho** | **8** | **-47.07** | **6.68** | **1** | **0.010** | **0.13 (0.04, 0.21)** |
| Rate of prey escape | size | 7 | -46.31 |  |  |  |  |
|  | size+tot.coho | 8 | -45.67 | 1.28 | 1 | 0.259 | -0.08 (-0.21, 0.05) |
| **Proportion of strikes successful** | - | 4 | -127.29 |  |  |  |  |
|  | tot.coho | **5** | **-124.56** | **5.46** | **1** | **0.019** | **0.08 (0.02, 0.15)** |
| Proportion of thefts successful | - | 4 | -37.72 |  |  |  |  |
|  | tot.coho | 5 | -37.24 | 0.94 | 1 | 0.331 | 0.10 (-0.11, 0.31) |

Notes: **df*: degrees of freedom of the model. † NLL: negative log likelihood. ‡ Test statistic for the Likelihood Ratio Test: $\chi^{2}=-2\ln\left( \mathrm{NL}L_{\mathrm{null}}/NLL_{\mathrm{size}} \right)$. § degrees of freedom for the Likelihood Ratio Test = *df*_size_ - *df*_null_. ‖ Slopes are on the scale of the linear predictor.

**Additional empirical observations**

## Methods

The objective of these additional observations was to have a smaller net pens where predatory behavior could be more easily and accurately observed, and smaller groups of coho that were clearly distinguishable in size, rather than from a more continuous size distribution as those in Study 1 (Fig. S1). We conducted ten 30-minute trials involving 10 coho each. The trials were run for 30 minutes instead of 60 minutes as in Study 1 because we observed that most of the predation behavior occurred at the start of the trials (Fig. S3). The second cohort of coho predators from the first observational study (*n*  = 111) were divided into four size groups: small (81-95 mm, *n=5*), small-medium (96-110 mm, *n=25*), medium-large (111-125 mm, *n=55*), and large (126-140 mm, *n=15*) so that small coho could be paired with medium-large coho (one trial) and small-medium coho could be paired with large coho (three trials). These size classes roughly correspond to the small, small, medium and large classes from the initial observations, respectively. These four mixed-size trials included five coho from each of the two size classes involved. The remaining six trials involved 10 coho from the same size lass (one trial with medium-small and five trials with medium-large coho). Each coho was used only once in these additional observations, leaving eleven of the available coho that did not participate in any of the additional trials.

To facilitate observations, we conducted these trials in small, white net pens (2.1 m × 1.4 m × 1.5 m deep). Coho were food-deprived for 48 hours before the start of each trial and placed in the experimental pen at least one hour prior to the trial. The trial began when 40 pink salmon prey were released into the experimental pen. We recorded observations of the five foraging-related behaviors from Study 1 (above) as well as two additional behaviors: (1) consumptions when a captured prey was completely consumed and was no longer visible, and (2) handling time recorded as the time from capture (by the final coho if prey was stolen) to consumption. If the prey was stolen or escaped, we also noted the handling time from capture until loss of prey, but these times were treated as censored data in the analysis (see Data analysis, below).

### ****

Figure S 3. The frequency of observed behaviors of coho salmon predators over increasing time elapsed during trials in Study 1.

### **Data analysis**

There were few individuals in some of the four size groups, so we combined data for small and small-medium coho into a small group (81-110 mm, *n* = 30) and data for medium-large and large coho into a large group (111-140 mm, *n* = 70). Due to the lower number of trials and shorter duration of these additional trials, we did not use GLMMs to analyze the data (as we did for initial observations in the main text) but reported the average number of observations ±95% bootstrapped confidence intervals for captures, theft attempts, and successful thefts. First, we compared the frequency of those three behaviors between the small and large coho in trials with predators from different size groups (*n* = 4 trials). Second, we compared the frequency of those behaviors between trials with predators from the same size group (*n* = 6 trials) and trials with predators from different size groups (*n* = 4 trials).

Finally, we used a parametric survival analysis assuming constant hazard to compare the handling time between small and large coho. If prey were stolen or escaped, the handling time was treated as censored data.

### **Results**

Despite considerable variability among trials, the results for the additional observations using coho from different size groups showed the same tendencies as results presented in the main text: compared to large coho, small coho captured more prey and were less successful in their theft attempts (Fig. S3a); there were a total of four successful thefts by large coho but no successful thefts by small coho in mixed-size trials. Small coho were more likely to lose prey to theft (0.15 vs. 0.05 per coho) or have prey escape (0.30 vs. 0.15 per coho) than large coho. The consumption rate during the 30-minute trials was higher for large coho than for small coho (0.70 versus 0.50 prey consumed per coho). Although these results are consistent with our initial observations presented in the main text, none of these additional results were statistically significant. The small number of trials was a major limitation and may have limited the statistical power of the results, as seen by the wide confidence intervals in Fig. S3.

The four trials with mixed-size coho showed fewer captures but more theft attempts and more successful thefts than trials with similar-size coho (Fig. S3b), though this was also not significant. This suggests that scrounging strategies are more likely to be adopted when the group includes individuals of different sizes.

The survival analysis of handling time showed that the size of the predator had a significant effect: handling time of captured prey was 89.8 s (95% CI: 57.2 s, 152.5 s) for small coho and 43.3 s (32.5 s, 59.6 s) for large coho. Thus small coho not only captured more prey but they also spent more than twice as long handling prey as large coho, making them inviting targets for scroungers.


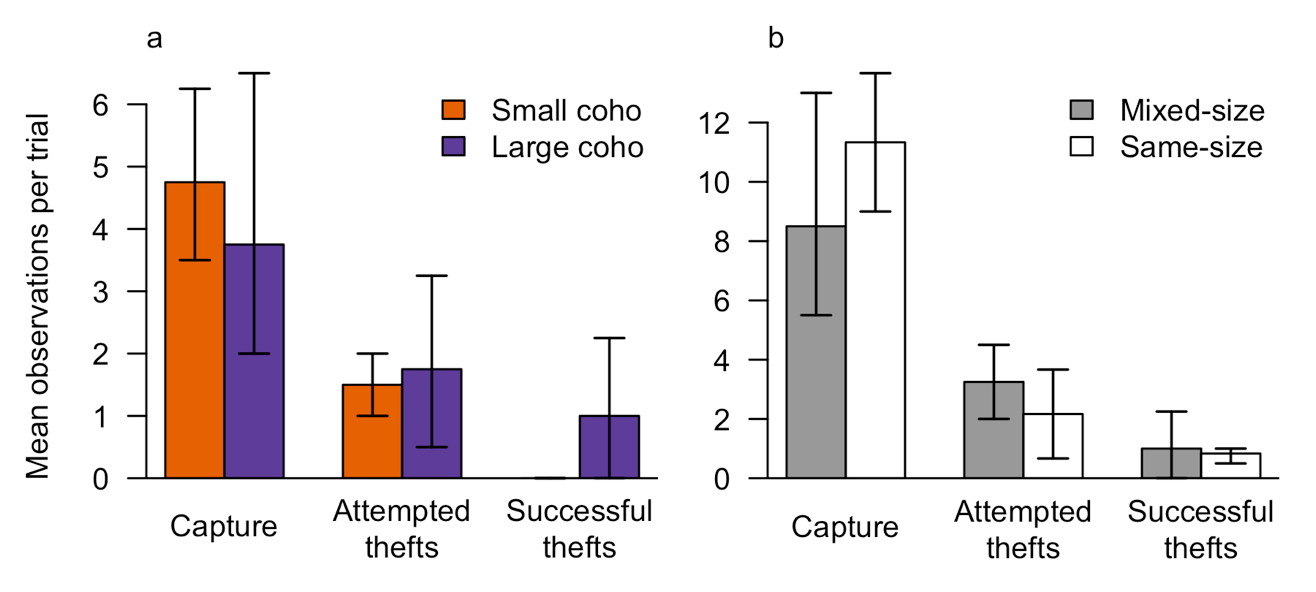


Figure S 4. Mean number of captures, theft attempts and thefts per trial for (a) small and large coho in mixed-sized groups (n = 4 trials) and (b) groups with both small and large coho (n = 4 trials) versus groups with the same-size coho (n = 6 trials). Error bars are 95% bootstrapped confidence intervals.
